# Psychological, social, and physical ecologies for child resilience: a South African perspective

**DOI:** 10.3389/fpsyg.2023.1190297

**Published:** 2023-07-25

**Authors:** Jace Pillay

**Affiliations:** Faculty of Education, University of Johannesburg, Johannesburg, South Africa

**Keywords:** Africa, child resilience, physical, psychological, social, socioecological

## Abstract

Children live in a complex world surrounded by global concerns such as climate change, economic instability, threats of terrorism and war. However, in South Africa, one may note that children face several additional challenges including high unemployment rates in families, exposure to violence, living in conditions of poverty, exposure to HIV/AIDS, and high levels of orphanhood. Compounding these problems is the economic situation in the country where the government is unable to provide adequate support for children in various domains. Understanding the mechanisms through which children successfully adapt to their environments and transition into adulthood are important to understand. Resilience research seeks to understand these mechanisms and underlying processes that enable some individuals to recover from adversity against all odds. Therefore, there is an increased movement not only toward understanding resilience processes in children, which enable them to develop into fully functional and upstanding citizens of society despite the adversities they face, but also how resilience research can be translated into practice to be used by service professionals such as psychologists, school counselors, social workers, and teachers. Adopting a socioecological understanding of resilience, the author reviews literature on the psychological, social, and physical ecologies for child resilience globally. Special emphasis is placed on the ecologies of child resilience within the African context and South Africa in particular. A socioecological perspective positions child resilience within four important levels, namely individual, relationships, community, and society. The salient features of child resilience within a South African context are discussed within the four levels highlighting the implications for interventions to promote child resilience. The implications have global value because child resilience is a phenomenon that needs global attention.

## Background and introduction

With an increasingly complex world that children are part of, including concerns such as climate change, economic instability, threats of terrorism and war (Masten, 2018), understanding the mechanisms through which children successfully adapt to their environments and transition into adulthood are important to understand (Norris and Norris, 2021). Resilience research seeks to understand these mechanisms and underlying processes that enable some individuals to recover from adversity despite the adversity (van Breda and Theron, 2018). Although numerous debates around the definition of resilience exist (Masten, 2018; Van Breda, 2018, p. 4) define it as “the multilevel processes that systems engage in to obtain better-than-expected outcomes in the face or wake of adversity.” In South Africa, youth face several challenges including high unemployment rates, exposure to violence, living in conditions of poverty, and high levels of orphanhood (Ebersöhn, 2017; van Breda and Theron, 2018). Compounding these problems is the economic situation in the country where the government is unable to provide adequate support, in various domains, to young people and there is great concern for their health and well-being (van Breda and Theron, 2018). Therefore, there is a need for professionals such as psychologists, social workers and school principals to develop and use resilience-based programs for children (van Breda and Theron, 2018).

At the forefront of this movement, Ungar and Theron (2020) advocate for a socioecological understanding of resilience, that is resilience as understood not only as intrinsic factors of the individual, but also factors that exist in the individual’s contextual realities including the cultural norms that influence their resilience processes. They argue that mental health researchers agree that “systemic influences matter at least as much as individual factors to positive outcomes” (Ungar and Theron, 2020, p. 441). They advocate for an understanding of resilience that includes the complex interactions of an entire biopsychosocial ecological system comprised of systems of one’s biology, psychology, social networks, built environment and natural environment. Similarly, Theron (2019, 2020) argues that resilience research needs to be sensitive to varied contexts of young people and how resilience processes may differentially impact young people depending on these unique contexts. Hatala et al. (2020, p. 10) argue along the same lines stating that “it remains crucial to understand youths’ resilience from a relational worldview that encompasses the context, the mental, emotional, physical and spiritual connections with land and nature, as well as the unique interactions and structural impediments to well-being and resilience.” In the South African context Ebersöhn (2017) argues that the health and well-being outcomes for young people in an unequal society are relevant to educational research. This means that the emphasis should be on improving psychological, social, and educational well-being.

Taking the above into consideration the literature review that follows focuses on a central research question: What are the psychological, social, and physical ecologies for child resilience in South Africa and the implications for child resilience interventions? The review is structured into three broad ecologies of resilience: psychological, social, and physical. Each part discusses the extant literature internationally, in sub-Saharan Africa (SSA) and in the South African context. It is imperative to note that all three categories are interactive and interdependent and not linear in function but are presented separately just for clarity in this paper.

### Methods

This study utilized a narrative review framework to gather published studies in the area of resilience in relation to children both in a local South African context as well as internationally.

### Search, selection, and review method

The protocol followed during the literature search was explicitly focused on published studies in academic journals. This focus was intentional in order to collate studies that focused on particular ecologies of child resilience. The primary databases used to source international literature were SCOPUS and Web of Science. Key words included in searches, in varying combinations, were “child resilience”, “mental health”, “social well-being”, “physical ecologies”, “socioecological resilience”, and “green spaces”. Search results were filtered to only include journal articles. Initially search results were limited to 2017 onwards, thus most of the literature reviewed is from 2017 onwards. For more local literature from Africa as well as South Africa, SCOPUS, Web of Science, and SABINET African Journals databases were used. The same keywords as listed above were used, with the addition of ‘South Africa’ when seeking South African literature specifically. Again, search results were filtered for journal articles only. The same date range was applied. For physical ecologies in South Africa, the date had to be extended as far back as 2008 due to limited articles focusing on that area.

In terms of selection criteria, article titles and abstracts were screened for relevance based on the topic. Three primary criteria were applied during the screening process: (i) the articles had to address at least one of the domains of resilience, namely psychological, social, ecological/physical resilience; (ii) articles had to be focused specifically on children or at least include children (where children were defined as younger than 18 years); (iii) articles had to be published in English (or have a translated version available) to be included as the researcher’s primary language is English. Due to the review being more of a narrative-type review, not all articles found were included. Rather, only those deemed by the research to be of most relevance to the topic were utilized. A brief reflection on the limitations of such a method are provided near the end of this article.

Selected articles were then read and categorized into either international, African or South Africa studies and within each context were further categorized into articles focusing predominantly on psychological resilience, social-based resilience, and ecological/physical resilience. Articles were then re-read, and primary findings extracted, collated, and written up, which forms the bulk of this article in the sections that follow.

### Psychological ecologies of resilience

At an *international level* Rasmussen et al. (2019) conducted a systematic review and meta-analysis on the relationship between secure attachment style and resilience based on 33 reviewed studies primarily from global north countries. Their findings indicate there is a statistically significant and positive correlation between the quality of attachment and the presence of resilience properties. They further argue that stable attachment relationships are an important factor in fostering resilience, which can help buffer against adverse outcomes in adult life. They also note that stable attachment does not necessarily have to be between a child and a primary caregiver, but rather family members, or teachers, who can also provide the same kind of secure attachment, which they term as “earned security”, that can help enable resilience in children (Rasmussen et al., 2019). Similarly, a study on young adults in the Unites States of America (USA) found more positive mother–child relationships in childhood predicted higher levels of resilience and higher levels of close attachment (Kennison and Spooner, 2020). Further, an interesting contribution of this study was that lower levels of negative father-child relationships predicted higher levels of resilience. They reported that social support and social skills were particularly important when examining the relationships between parent–child relationships and attachment.

Similar links between attachment and resilience have been found in China too. A study based on 284 impoverished Chinese middle-school students examining mother–child attachment, resilience and psychological needs satisfaction found that mother–child attachment was positively related to psychological needs satisfaction. Positive needs satisfaction was then positively related to resilience, thus showing a mediated relationship (Wang et al., 2020). This finding is important as it suggests that despite economic hardship, fostering attachment between mother and child can help buffer against poor outcomes for the child. Quality of mother–child attachment was also positively associated with resilience; thus, the mother–child relationship is protective for child resilience (Wang et al., 2020). A study was conducted on children from low-income families in China based on the relationship between shyness and resilience on parent–child attachment and teacher-student relationship (Wang et al., 2022). It was reported that resilience moderated the relationship between shyness and teacher-student relationships. This relationship was stronger for children with low resilience as compared to their high-resilience counterparts (Wang et al., 2022). This suggests that a shy child may still be able to cope well in social situations if they are resilient, thus showing how resilience is a positive adaptive mechanism. Shyness was found to partially mediate parent–child attachment and teacher-student relationships, where shyness was negatively related to parent–child attachment. This suggests, in line with attachment theory, that more securely attached children exhibit less fear and anxiety in social situations (Wang et al., 2022).

A study based in Iran comparing working and non-working children on measures of stress-coping strategies, resilience and attachment styles indicated that, as expected, working children were more likely to have lost one or both parents, thus forcing them into work and consequently keeping them out of education (Pasyar et al., 2019). Working children reported lower levels of resilience compared to non-working children in this study, indicating that child labor hampers the development of resilience. These children often face less opportunities to develop resilience hence this finding is unsurprising (Pasyar et al., 2019). The physical environments working children find themselves in, such as impoverished streets and workshops, no doubt impact as risk factors and less the chance of developing resilience (Pasyar et al., 2019).

Children living in *Sub-Saharan Africa* (SSA) face numerous adversities, including communicable diseases, pervasive poverty, inequality, and armed conflict, among others (Theron, 2020). A narrative scoping review of research on the resilience of youth in 18 SSA countries observed the complex and multifarious nature of resilience in children and adolescents (Theron, 2020). The results indicated two primary findings: firstly, that personal support mechanisms and relational support mechanisms seemed to matter equally for young people; and secondly, the capacity to adjust positively to life challenges has a complex relationship with African ways-of-being and ways-of-doing. Interestingly, this review found no references to structural resources from school systems (Theron, 2020). Theron (2020) also suggests that, in-line with a collectivistic and relational worldview generally adopted by Africans (Bojuwoye and Moletsane-Kekae, 2018), that practitioners be appreciative of the complexities between relational and internal resources of young people. Theron (2020) also cautions against seeing resilience processes as universal, as some studies reviewed showed that individual agency was not consistently protective for young people.

A mental health intervention in Tanzania targeting youth living with HIV was conducted by Dow et al. (2018). The primary aim of the intervention was to increase the resilience of these young people. The study reported that the intervention was successful, with utility noted for the use of narrative in the intervention, which was associated with trauma reduction, normalizing experiences among peers and promoting interpersonal communication (Dow et al., 2018).

Zooming into *South Africa* (SA) a systematic review by van Breda and Theron (2018) was conducted on youth resilience studies of children between 2009 and 2017. They reported numerous adversities, which were primarily structural such as poverty and living in under-resourced communities. Other adversities also included being HIV positive, orphanhood, experience of violence, sexual abuse and being a refugee. In their content analysis of the articles reviewed, they reported that personal or relational resilience-enablers were most frequently reported, which included aspects such as teacher support, educational aspiration, financial well-being, and community support and safety. Although not a primary finding, adherence to African values such as *ubuntu* were also found as helpful in promoting resilience. Ubuntu refers to a collection of values and practices that African people have that makes them authentic individual human beings as part of a greater collective world (Mugumbate and Chereni, 2020) Affective support was found as the most reported factor specifically, encompassing feelings of being valued and a sense of belonging. This was primarily through friends, parents, caregivers, and teachers. The interaction of resilience-enablers was uncommon. van Breda and Theron (2018) recommend that practitioners understand the interaction of resilience-enablers and help make young people aware of additional resources available that would fit well with their current resources to bolster their resilience.

A multicounty study, of which South Africa was part, on trauma, resilience and mental health in migrant and non-migrant youth reported that South African adolescents had the highest mean number of traumatic events over the past year compared to the other five countries who took part in the study (Gatt et al., 2020). Interestingly, the study found that migrant adolescents had higher levels of resilience resources compared to non-migrants, despite migrants experiencing more traumatic events. Further, the impact of traumatic events on adolescent mental health was higher for non-migrants (Gatt et al., 2020). However, the authors argued that further research needs to be done to investigate how resilience can be promoted in youth irrespective of them being a migrant or not. A randomized clinical trial testing the efficacy of an intervention aimed at improving the resilience of young children with HIV-positive mothers in Tshwane, South Africa reported promising results (Eloff et al., 2014). The intervention group reported significant improvements in the domains of their children’s externalizing behaviors, communication and daily living skills. Internalizing behaviors and socialization improvements were not significant, however. The results of the study also suggested that boys benefitted more than girls, but the authors argue that these differences likely have little practical relevance as both boys and girls still benefited from the intervention (Eloff et al., 2014).

Theron et al. (2022) performed a study on 21 South African and 31 Canadian youth who live in stressful environments. They reported that within the South African youths’ psychological systems, future-oriented agency and seeking and reciprocating help were key processes that emerged. In contrast, the Canadian youth reported self-regulation and self-efficacy in their psychological systems. For the South African youth, it was observed that their biological, psychological, and informal social resources interacted. As an example, able-bodied young men spoke about how physical strength (biological domain) mattered in the context of seeking out manual labor (economic domain), while young women pushed back against gender stereotypes (social domain) letting this drive their agency while simultaneously finding inspiration from role models in their community (intra and interpersonal domains). South African youth also expressed how striving for economic independence was associated with the benefits this would bring to psychological well-being (Theron et al., 2022). Additionally, Theron et al. (2022) reported that psychological and social system support emerged the most in their data, however they advocate for a multisystemic approach to understanding resilience in youth more holistically, in line with Ungar and Theron’s multisystemic approach Ungar and Theron (2020).

### Social ecologies of resilience

In an *international* study on practitioners’ perspectives of caregivers’ influence on the development of resilience in maltreated children it was found that practitioners believed that maintaining a stable home environment characterized by consistency, predictability and safety were essential in promoting resilience (Beaujolais et al., 2021). Specific behaviors that enable resilience, according to the practitioners, included prioritizing the needs of the child, believing disclosures made by the child and verbalizing belief in them. Furthermore, the participants advocated for a systems-based approach where family involvement in the child’s life was important (Beaujolais et al., 2021), an approach supported by researchers too (Twum-Antwi et al., 2020). Unsurprisingly, the participants also indicated that a caregiver who is also a perpetrator of child maltreatment is a large barrier to the development of resilience in these children. An investigation of coping strategies used by undocumented Mexican youth in the USA argued that family-level coping strategies included parents providing informational support and emotional support (Kam et al., 2018). Informational support was utilized to protect the family unit, such as information on how to avoid deportation. Emotional support emerged as a means to safeguard the family’s positive future by working together as a family unit to manage their lives, contribute to the household economics and support each other through shared stressors. This research also indicated that parents sometimes avoid discussing their undocumented status with their children to shield them from the potential stress this may cause (Kam et al., 2018).

Along similar lines of the ways that youths’ social ecologies can promote resilience, Twum-Antwi et al. (2020) investigated the ways in which child and youth resilience can be strengthened by home and school environments. They argue that in the home factors such as parents’ mental well-being, self-efficacy, parenting satisfaction and parental confidence are key factors in the outcome of parent–child relationships. Parental resilience, which consequently has an impact on child resilience, is fostered by the support of family, friends, and community resources. Similarly, within a school environment, teacher mental well-being, feelings of self-efficacy and job satisfaction are important in fostering the relationship between the teacher and the child (Twum-Antwi et al., 2020). Thus, the authors conclude that “educators and caregivers should not only make efforts to help children; they need to also help themselves” (Twum-Antwi et al., 2020, p. 84), thus demonstrating the systems-based approach that several researchers advocate for (van Breda and Theron, 2018; Theron, 2020; Ungar and Theron, 2020; Norris and Norris, 2021).

Glaser et al. (2022) conducted a study on the impact of a physical activity online intervention program during the COVID-19 pandemic on the resilience levels of 56 secondary school youth in Israel. They reported that pre-test resilience levels of the intervention group were lower than the control group but were equal to the control group post-test. The intervention improved participants’ resilience, feelings of social support and decreased their levels of psychological distress (Glaser et al., 2022). Other studies have suggested similar advantages of physical activity for resilience development. Norris and Norris (2021) discuss the potential that being involved in sporting activities, and physical activity more broadly, can have on the physical and mental health of children where such benefits may be associated with resilience and thus can act as a buffer against adverse childhood experiences. Belcher et al. (2021) review article examined the links between physical activity and fitness and their impact on resilience in adolescents by examining changes in self-regulation. They argued that physical activity is linked to both structural and functional changes in both cognitive and emotional systems in the brain associated with mental health, thus suggesting that physical activity may be something that can help foster resilience and consequently mental health. Along similar biological lines, Niitsu et al. (2019) reviewed studies on the genetic influences of psychological resilience, reporting that six genes were associated with psychological resilience. However, they cautioned that such results are complex and involve intricate interactions of genes on resilience.

*Sub-Saharan Africa* faces numerous challenges, including high incidences of climate-related disasters (Bakshi et al., 2019), violent conflict (Allansson et al., 2017), high rates of gender-based violence (Muluneh et al., 2020), and high levels of trauma exposure (Ng et al., 2020). Thus, fostering socioecological resilience in these countries is essential to help buffer against the deleterious outcomes of the citizens in these countries, especially the youth. Faith (also referred to as spirituality and religion) can be an important aspect in the development of resilience in youth (Mhaka-Mutepfa and Maundeni, 2019). Mhaka-Mutepfa and Maundeni (2019) argue for more studies that assess the role of faith in resilience development longitudinally. They advocate for policy-level interventions where governments of SSA countries encourage religious bodies and traditional leaders to put steps in place to support the development of children. They note that aspects such as hope, love and forgiveness, key elements as part of faithful practice, may enhance children’s well-being and resilience (Mhaka-Mutepfa and Maundeni, 2019). A study investigating the relationship between religion and resilience in youth was conducted by Gunnestad and Thwala (2011) in Zambia and Swaziland. Their analysis revealed that participants used religion as a resource to help handle life challenges. Religion provided connection with peers and role models, hope from their faith, and faith-based counseling to help cope with life difficulties (Gunnestad and Thwala, 2011).

While faith-based resilience has shown utility, other social interventions such as sport have also shown promise in SSA. Malete et al. (2022) examined the effects of a sports-based intervention program on the life skills and entrepreneurial development of youth from Botswana, Ghana and Tanzania. Post-test scores suggested that the sports-based intervention significantly increased the life skills of the participants. This suggests, along the lines of other research studies in international contexts (White and Bennie, 2015; Fader et al., 2019), that sport is a valuable tool that can be used to intervene in the lives of young people with the objective of increasing their capacities to deal with life’s challenges (Malete et al., 2022). A study based in northern Uganda explored the relationship between resilience, ideas of morality and community well-being through the lens of sport (Abonga and Brown, 2022). Young people in this study believed that sport was important to their development of resilience aiding in their acquisition of physical, material, and emotional resources, but it was noted that sporting programs need to be sustained. These researchers do acknowledge the challenges in resilience research, pointing out that sports program may reinforce inequalities such as dominance and patriarchy (Abonga and Brown, 2022). They argue that “programming for resilience, even culturally sensitive programming, will face this challenge if it fails to nuance the understanding of whose vision of resilience it is trying to promote and what elements of society it is trying to reinforce” (Abonga and Brown, 2022, p. 249). Craig et al. (2019) argue from a Malawian context that sport, as a form of therapeutic recreation for disabled youth, can be leveraged to empower young people and increase health outcomes. They discuss that in Malawi there is currently a diverse and dedicated group of sports leaders, however they do not have a central organizing network or have best practice guidelines to help them reach more young people (Craig et al., 2019).

Pswarayi (2020) conducted a qualitative study in Zimbabwe, using both focus groups and individual interviews, to examine the relationship between youth resilience and violence prevention. Despite the very trying socio-political climate in Zimbabwe characterized by pervasive poverty and high youth unemployment rates, the participants demonstrated how they utilized various processes of resilience, such as adapting to their environment and managing the pressures of their environment, in order to function effectively. Participants indicated how they were able to leverage their networks in order to link them to opportunities in the informal sector. Pswarayi (2020) notes that in Zimbabwe, there has emerged an alternative economy, the *kiya-kiya* economy which refers to making ends meet as a means of survival for young people, which is largely unregulated and informal and includes gambling spots. Pswarayi (2020) argues that the *kiya-kiya* economy reflects resilience of young people as it demonstrates their adaptive capacity to their context. However, sometimes this involves acts of criminality and violence to defend these informal economies.

A Kenyan study examining resilience and dialog with 120 participants, of which 30 were youth, reported that violent extremism in the country is a major problem resulting in human rights violations, discrimination, and socio-economic struggles (Sigsworth et al., 2020). The results of the study indicated that community resilience does exist as evidenced by the integration between differing identity groups and the coordination between people in helping solve shared problems. Participants noted that dialog between communities and law enforcement, and within communities themselves, is a barrier to peace at time. However, participants identified several areas where resilience can be fostered including maintaining cultural/religious identities, strengthening social cohesion, and including the voices the marginalized. It is the resilience of these participants that needs to be supported to continue the growth of dialog among people of differing creeds in order to work collaboratively toward a better and safer future in Kenya (Sigsworth et al., 2020).

For *South Africa*, Höltge et al. (2021) reported that the importance of getting an education was one of the top central resilience resources. Mampane and Bouwer (2011) argue that learners living in South African townships require resilience to overcome the various adversities they face. Their qualitative study on two township schools with learners reported that the influence the schools had on the learners varied according to their degree of resilience but was also influenced by the factors within the schools (Mampane and Bouwer, 2011). Factors such as providing life skills and clear rules of conduct were important to some of the participants. This suggests that schools as social ecologies have an important role to play in the development of youth resilience, where the influence of the school extends well beyond the provision of knowledge alone. The study also found that goal attainment seemed to have a strong relationship with resilience (Mampane and Bouwer, 2011).

A study conducted in the Free State with teachers from 20 schools used the Circle of Courage model for teachers to help build resilience (Reyneke, 2020). This model uses strengths-based techniques to encourage the development of resilience in learners by enhancing their sense of belonging, mastery, independence, and generosity. The study reported that the teacher participants needed to improve in all aspects of the model, particularly mastery, which was reported as the lowest scoring technique across the sample (Reyneke, 2020). Reyneke (2020) argued that increased use of the Circle of Courage framework and the development of the techniques the model outlines will help teachers improve their learners’ resilience.

Bhana and Bachoo (2011) argue in their systematic review that family resilience is related to factors such as family cohesion, health belief systems, good parenting practices and social support. They argue that, in impoverished contexts specifically, community and social support plays a salient role in developing family resilience (Bhana and Bachoo, 2011). A quantitative investigation into the association of psychosocial vulnerability and family resilience with academic achievement in primary school learners in a township in Gauteng, South Africa, found that there was only partial support for the hypothesis that higher levels of family vulnerability are associated with lower academic achievement (Van Breda, 2022). Similarly, partial support was noted for learners with higher levels of individual or family resilience having higher academic achievement where this relationship only held for learners who had experienced more challenging life events. Although this study also offered a family-strengthening intervention, it did not improve academic achievement (Van Breda, 2022). These findings were surprising as it is generally believed that increased family strength positively impacts academic achievement and so schools and communities are encouraged to try strengthening resilience in families (Van Breda, 2022). It is for this reason that family-based interventions are designed, as in the case of Isaacs et al. (2018). Their contextually based family resilience program was designed for families in rural South African communities. They argue for the importance of the contexts that such programs are designed for and advocate for evidence-based research to be used in the designing of such interventions (Isaacs et al., 2018). Their intervention contained four modules including “about family”, “closer together”, “talking together”, and “working together” – all elements that aim to increase connectedness, communication, and social and economic resources. Further, they emphasized that although family-based resilience-promoting interventions may help improve family life, “it especially does not preclude the rights that all individuals have to be protected from structural adversity” (Isaacs et al., 2018, p. 633). Hence, government’s role in assisting its citizens must always necessarily be an active one.

### Physical ecologies

In an *internationa*l cross-country study on the interaction between social and ecological resources on adolescent resilience Höltge et al. (2021) found that all 14 countries reviewed had different resilience networks, pointing toward how unique environmental contexts influence resilience. While psychological and social determinates of resilience are important, the physical environment in which youth find themselves embedded may also impact on development of resilience processes. Ungar (2017, p. 1280) succinctly notes that research often acknowledges “the link between neurons and neighborhoods, or cells and communities” through person-environment interactions. Thus, it is equally important to understand the physical ecologies that may impact youth resilience (Ungar and Theron, 2020). Studies have shown that neighborhoods with higher social cohesion can act as a preventative factor against maltreatment in children (Abdullah et al., 2020), more socially cohesive neighborhoods have been associated with buffering against the deleterious effects of stressful life events helping keep depression/anxiety, suicidal ideation and aggressive conduct levels lower in youth (Kingsbury et al., 2020), and higher levels of neighborhood support have been shown to help reduce aggression and delinquency in youth exposed to violence (Jain and Cohen, 2013). A qualitative study in the US exploring the environmental health perspectives of urban youth reported that the theme of environmental health resilience factors was constructed in their study based on their focus group data, with subthemes of safety, trust, engagement, leadership, and representation emerging (Bogar et al., 2018). They argue that safety and trust are environmental health resilience factors that enable youth to navigate their local spatial contexts, where youth develop “cognitive risk maps” of which areas are safer (such as community gardens) and which are not (such as areas frequented by discriminatory law enforcement officers) (Bogar et al., 2018). They further note how race or power differentials, such as differing socioeconomic statuses, play their role as structural drivers of which spaces are more accessible and safer for youth, which consequently has an impact on their environmental health resilience (Bogar et al., 2018).

There has been a growing interest in the relationship between humans and their natural environment and the consequences of this relationship for health (Seymour, 2016). Studies have shown that for children spending more time in nature is related to health development, well-being, and positive attitudes toward the environment (Gill, 2014). Similar findings have been noted for adults where Wood et al. (2017) reported that the total area of public green spaces was associated with increased mental well-being, with a dose–response relationship being noted. Thus, the potential positive impact of the natural environment on mental health needs to be taken seriously. Hatala et al. (2020) explored the meaning-making practices of Indigenous Canadian youth using photovoice regarding their perceptions of the environment and its influence on their health and resilience. Their study reported that participants constructed nature as a calming place, using metaphors such as those of the seasons as linked to cyclical phase of life, and further inscribed a sense of hope into their natural environment. Participants linked their experiences of the natural environment to their coping with stress, fear, anger and general difficulties in their daily lives. This study contributes to the growing body of knowledge that has begun to acknowledge how nature or land plays a role in supporting youth resilience and well-being (Hatala et al., 2020).

In an *African* study it was found that physical space, and the safety of that space, influences the ways in which people adapt to their environments and it has an impact on how the built environment is shaped (Watson, 2009). Watson (2009) argues that urban planning has traditionally excluded the poor. One need not look further than urban spatial planning in apartheid South Africa for examples of this, where to this day cities across the country still echo constructed divisions of the past (Maharaj, 2020). Oosthuizen and Burnett (2019) investigated how residents in an impoverished township in Johannesburg perceived their use of spaces and how they view these spaces in terms of safety. Their sample consisted primarily of learners from local schools. From their findings, they generated four typologies of space based on safety and activity: unsafe activity supportive, safe activity supportive, unsafe activity unsupportive and safe activity unsupportive. The authors argue that such community mapping is useful so that schools and NGOs can identify safe spaces where they can implement sporting and physical activity programs for youth to increase participation (Oosthuizen and Burnett, 2019). The development of such spaces may aid in providing networks of activities that can promote youth resilience (Ungar and Theron, 2020).

Studies have also provided evidence of the impact one’s physical neighborhood can have on mental health and resilience. A South African study assessing the relationship between social capital and youth mental health utilizing family social capital and neighborhood social capital as its primary variables outlined that family social capital, as measured by household income, decreased the odds of depression, while higher perceptions of crime in the participants’ neighborhoods increased the chances of depression (Somefun and Fotso, 2020). These findings suggest that although increased family social capital is associated with decreased mental illness, it does not necessarily promote increased mental well-being (Somefun and Fotso, 2020). A study based in Reservoir Hills in Durban, South Africa, a historically Indian middle-income area, examined community member perceptions of safety regarding urban open spaces (Perry et al., 2008). Parks and open spaces in the neighborhood were viewed as unsafe by participants, who felt fearful of these spaces due to potential criminal activities, regardless of whether the spaces were well-maintained or not (Perry et al., 2008). This is in contrast to research more globally which generally indicates more green space is associated with lower incidences of illegal activity in urban environments (Shepley et al., 2019). These contrasts echo Ungar and Theron’s (2020) arguments that resilience has to be understood contextually and locally produced, hence no two ecological systems are the same.

## Implications for child resilience interventions

The preceding discussion provided an understanding of the social, psychological, and physical ecologies of resilience which I consider essential to be positioned within a socioecological perspective. This perspective is feasible because it provides an understanding of resilience on four levels, namely individual, relationships, community, and society (Dahlberg and Krug, 2006). The interaction between children, their relationships with others, community exposure, and societal factors have an integrative and holistic impact on their levels of resilience (Edberg et al., 2017). As such, resilience must be viewed as a dynamic and interactive ecology. At the individual level children’s biological and personal history (age, family income, and education) contribute to their resilience. At the relationship level children’s interaction with family and community members as well as peers at school can contribute to their levels of resilience. At the community level, schools, local neighborhoods, and religious organizations could also influence resilience in children. At the societal level several factors can impact the resilience of children, for example, social, and cultural beliefs and practices and policies that promote economic and social inequalities among people. The four levels are not mutually exclusive but are interdependent in promoting or reducing resilience in children. Resilience does not result from a single level but from multiple systemic influences that impact all four levels. Taking these levels into consideration I now point out the implications for child resilience interventions within a South African context.

At the *individual level* the psychological ecology of resilience is crucial. Virtually all literature notes that attachments between children and parents, irrespective of different contexts, seems to be an important determinant of child resilience, especially in reducing anxiety and fear in children (Wang et al., 2022). However, in a Western and Eurocentric context prominence may be given to the psychological constructs of self-regulation and self-efficacy to promote individual agency of children but in an African context interaction with dispositional characteristics and social resources appear to support the psychological empowerment of children (Theron et al., 2022). Being valued by others and having a sense of belonging is important for child resilience within an African context. A wisdom for child psychological resilience from Africa is attachments with parents/caregivers are important but attachments with others (peers, teachers, and community members) are just as valuable, for example, many orphans in South Africa succeed in life by adopting positive role models that make them believe in a better future and being open to help from others. Essentially parents, family members, peers and teachers play a pivotal role in positively contributing to psychological ecologies of resilience in children. A loving, caring, stable, and mentally healthy parent/caregiver could psychologically empower children. In an African context this can even be a family or community member, a teacher, or a religious leader since family is viewed in a broader context. As such, we see an interaction with psychological and social ecologies that can contribute to child resilience. At an individual level physical ecologies are also important for child resilience, for example, literature cited earlier have shown that social cohesion in local communities is linked to reduced levels of stress, depression, anxiety, and suicide attempts (Abdullah et al., 2020; Kingsbury et al., 2020). Exposure to natural environments is known to have a positive impact on mental health of children (Somefun and Fotso, 2020). However, in South Africa children are not encouraged to go to parks and gardens due to safety and security issues. Physical activities and sport also have a positive effect on psychological wellbeing (Oosthuizen and Burnett, 2019) so these should be included in school programs.

At the *relationships level* the literature reviewed indicates that positive, healthy, and close relationships contribute to child resilience. The relationships children develop with their parents/caregivers, family members and peers are crucial in them becoming resilient. Resilience-based strategies at this level should focus on positive parenting and family relationship skills, parent–child communication skills, mentoring, and peer education programs. In an African context children depend on both individual and collective agency to be resilient even though the latter may be more dominant. One of the wisdoms in an African context is that kinship ties are strong, so children are cared for by other family members if their parents are deceased. However, economic hardships are eroding kinship ties (Pillay, 2020).

At the *community level* characteristics of schools and local neighborhood settings play a vital role in child resilience. Safe schools and neighborhoods are most likely to build psychological, social, and physical resilience in children (Nitschke et al., 2021). Community and religious-based organizations can be instrumental in building resilience in children. Even in poor communities if there are facilities for children to learn, play and interact with others the probability of building resilience in them is likely to be greater. Schools are valuable institutions in the community because children spend a considerable amount of time at schools. This means that school management teams, school governing structures and teachers are strategically placed to address resilience-based programs as part of the school curriculum. The literature review pointed out the value of faith-based education and religious beliefs and practices in promoting child resilience. Despite poverty, crime, orphanhood, and dysfunctional families children still had positive future aspirations because of the faith they had in God (Asante, 2019). The wisdom from this is that faith-based education should be included in the school curriculum and places of worship.

The last level focuses on broad *societal facto*rs that contribute to child resilience. These factors are usually linked to social and cultural beliefs and practices that either diminish or support child resilience. For example, child labor and child marriages in some African contexts erode resilience in children. The blurred boundaries between cultural child rearing practices and corporal punishment are another example of reducing child resilience. At this level it is vital to determine how health, education, social and economic policies contribute to child resilience. Such policies in the apartheid South Africa maintained social and economic inequalities that often led to deleterious consequences for children and families negating child resilience. Promoting child resilience at the societal level should be directed at addressing the social and economic inequalities that exist in society, for example, the distribution of social grants for the unemployed, single parent and child-headed households, and free health and education facilities for children from poor homes and communities.

## Limitations

Limitations of this article primarily center around the type of review employed. While narrative reviews are useful to gather information about a subject field (Kastner et al., 2012), they lack the same rigor as other types of reviews such as systematic reviews. In this study, not all search results were screened as this was practically challenging due to the number of results produced, specifically for international literature. The author acknowledges this limitation. Further, the timeframe, especially for South African literature, was not as rigorously applied and had to be extended to prior to 2017 in order to include studies on all three domains of resilience examined. The study would also have benefitted from the inclusion of another researcher who could have screened articles as well so that the selection process was more rigorous. However, despite these limitations, the author is of the view that such narrative-type reviews hold value in reviewing a particular field of inquiry to understand the current trends and state of the research in said field.

## Conclusion

A comparison between child resilience within an African and Western context was not an aim of this paper but the literature review provided some common as well as very distinct differences in relation to child resilience within an African and Western context that should be noted. Common elements relate to secure parent–child attachments, safe, and nurturing environments. However, in an African context, interventions to promote child resilience places more emphasis on the external role of caregivers, families, and local communities while in a Western context the main focus is on the internal focus of control. This means from an African perspective child resilience will depend more on the people and society they interact with. Child resilience interventions must take psychological, social, and physical ecologies into consideration but more importantly interventions should be embedded within individuals, relationships, communities, and society. Interventions should include not just parents and family but also teachers, religious leaders, and community-based organizations. Faith-based education, culture, and future aspirations are crucial elements for African child resilience programs.

## Author contributions

The author confirms being the sole contributor of this work and has approved it for publication.

## Funding

The author (s) disclosed receipt of the following financial support for the research, authorship, and/or publication of this article. This work is based on the research supported by the South African Research Chairs Initiative of the Department of Science and Innovation and the National Research Foundation of South Africa (Grant Number: 87300).

## Conflict of interest

The author declares that the research was conducted in the absence of any commercial or financial relationships that could be construed as a potential conflict of interest.

## Publisher’s note

All claims expressed in this article are solely those of the authors and do not necessarily represent those of their affiliated organizations, or those of the publisher, the editors and the reviewers. Any product that may be evaluated in this article, or claim that may be made by its manufacturer, is not guaranteed or endorsed by the publisher.
